# Humidity-Mediated Anisotropic Proton Conductivity through the 1D Channels of Co-MOF-74

**DOI:** 10.3390/nano10071263

**Published:** 2020-06-28

**Authors:** Ali Javed, Ina Strauss, Hana Bunzen, Jürgen Caro, Michael Tiemann

**Affiliations:** 1 Department of Chemistry, Paderborn University, 33098 Paderborn, Germany; michael.tiemann@upb.de; 2 Institute of Physical Chemistry and Electrochemistry, Leibniz University Hannover, 30167 Hannover, Germany; juergen.caro@pci.uni-hannover.de; 3 Institute of Physics, University of Augsburg, 86159 Augsburg, Germany; hana.bunzen@physik.uni-augsburg.de

**Keywords:** metal-organic framework, Co-MOF-74, single crystal, impedance spectroscopy, proton conductivity, anisotropy, fuel cell

## Abstract

Large Co-MOF-74 crystals of a few hundred micrometers were prepared by solvothermal synthesis, and their structure and morphology were characterized by scanning electron microscopy (SEM), IR, and Raman spectroscopy. The hydrothermal stability of the material up to 60 °C at 93% relative humidity was verified by temperature-dependent XRD. Proton conductivity was studied by impedance spectroscopy, using a single crystal. By varying the relative humidity (70–95%), temperature (21–60 °C), and orientation of the crystal relative to the electrical potential, it was found that proton conduction occurs predominantly through the linear, unidirectional (1D) micropore channels of Co-MOF-74, and that water molecules inside the channels are responsible for the proton mobility by a Grotthuss-type mechanism.

## 1. Introduction

The predicted increase of the world’s energy consumption until 2050 is expected to be almost 50%, due to a higher industrial output, e.g., in the food or metal production sector, which comes with economic growth and an increasing world population [1]. Therefore, there is a rising demand for more efficient and sustainable energy conversion approaches. In this context, fuel cells, which allow the direct conversion of chemical energy into electrical energy, have been studied within the last few decades [2,3,4,5,6,7,8]. The most frequent type of fuel cells is the proton-exchange membrane fuel cell (PEMFC), in which protons are transported from the anode to the cathode through a proton-permeable membrane. The latter is usually made of Nafion, a sulfonated fluoropolymer, or of ionic polymers, which all suffer from high cost and only moderately efficient performance [9,10]. A promising approach to improve fuel cells is the incorporation of proton-conducting crystalline porous materials, such as metal-organic frameworks (MOFs) or coordination polymers, to replace amorphous polymers [11,12,13,14,15]. MOFs are hybrid inorganic–organic compounds, which have been vastly explored for more than two decades. Due to their modular design at the molecular level, a large number of MOFs (close to 70,000 already in 2017 [16]) exist nowadays; they can be further tailored by post-synthetic modification [17]. Their structural characteristics, such as high porosity, large specific surface area, controllable pore size, and well-defined channel systems, open doors to many potential fields of application [18,19,20,21,22].

**Figure 1:**
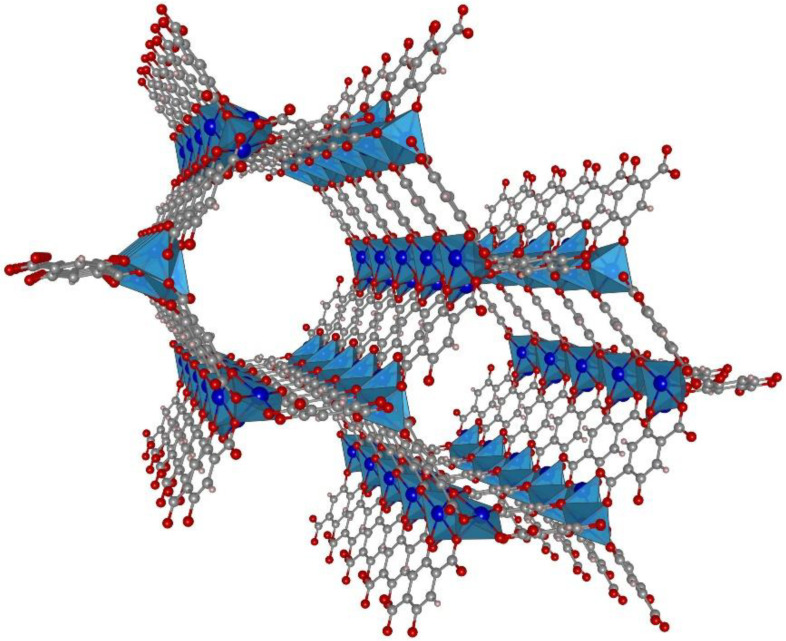
Crystal structure representation of Co-MOF-74 (Co—blue, C—grey, O—red); view along the crystallographic *c* axis. The structure is shown for the solvent-free state, i.e., the Co centers are penta-coordinated; under ambient conditions (humid air), the sixth coordination sites will be occupied by water ligands. (Crystal structure data taken from ref. [23], CCDC 270293; drawing made with VESTA software [31]).

Co-MOF-74, also known as CPO-27-Co, consist of Co(II) ions that are connected by 2,5-dioxido-1,4-benzenedicarboxylate linkers (Co_2_(dobdc)) [23,24]. The crystal structure (trigonal space group 148, *R*-3) is marked by linear micropores (1.1 nm) with a hexagonal cross section that runs parallel to the *c* axis of the crystals, as verified by sorption uptake studies with in-situ IR microscopy detection [25] (see Figure 1). The Co(II) ion is penta-coordinated by the linkers, and its sixth coordination site is exposed to the interior of the pore channels, usually coordinated by a water molecule. These structural features make Co-MOF-74 a highly interesting system with respect to host–guest interaction, e.g., for catalysis [26], gas adsorption [27,28], or gas sensing [29]. Moreover, water-mediated proton conduction is possible through the channels [30]. Here, we present a fundamental study on the proton conductivity of Co-MOF-74 for potential future utilization in proton-exchange fuel cell membranes. By applying impedance spectroscopy to a single crystal specimen, we investigate the anisotropy of proton conductance, as well as the impact of relative humidity.

## 2. Materials and Methods

Co-MOF-74 crystals were prepared according to a slightly modified procedure previously published by one of our labs [29]. A total of 750 mg cobalt nitrate hexahydrate (Co(NO_3_)_2_·6H_2_O, 99% Sigma-Aldrich, Taufkirchen, Germany) was dissolved in a 60 mL mixture of EtOH, DMF and H_2_O (1:1:1); afterwards, 144 mg 2,5-dihydroxy-terephthalic acid (DHBDC, 98% Sigma-Aldrich, Taufkirchen, Germany) was added. The suspension was ultrasonicated until the solids were dissolved completely, and subsequently heated to 131 °C for 24 h in a 60 mL Teflon-lined autoclave (Parr Instruments, Fankfurt, Germany). After cooling down to room temperature, the resulting crystals were washed and solvent-exchanged with MeOH. Then, the crystals were dried under reduced pressure and activated under vacuum at 160 °C overnight.

Scanning electron microscopy (SEM) images were obtained with a JEOL JSM-6700F NT microscope at 2 kV acceleration voltage and an emission current of 10 µA. IR spectra of a powder sample were measured with an Agilent Technologies Cary 630 FTIR spectrometer. Raman spectroscopy of single crystals was performed with a Bruker Senterra Raman spectrometer, and a laser excitation wavelength of 532 nm. Temperature-dependent X-ray powder diffraction (XRPD) data at relative humidity of 93% were recorded on a Panalytical Empyrean diffractometer, equipped with a CHC plus+ chamber in a transmittance Bragg–Brentano geometry, employing Cu-radiation. The patterns were recorded in a temperature range of 20–60 °C in 5 °C steps (each followed by a 15 min isothermal step), in the 5–50° 2θ range.

Impedance measurements were carried out with a Solartron SI 1260 Impedance/Gain-Phase Analyzer and a Novocontrol Alpha-A Analyzer. An experimental setup, as described previously by one of our labs, was used [32]. In short, one individual single Co-MOF-74 crystal was placed on an array of interdigitated Pt electrodes (20 μm electrode width and spacing; UST GmbH, Geratal, Germany) inside a custom-built Faraday cage that was placed in an Espec SH-242 climate chamber. The contact area between the crystal and the electrodes was estimated by confocal laser microscopy (Olympus LEXT OLS 3100, Hamburg, Germany), as shown in the Supplementary Materials section (Figure S1). The humidity was controlled by streaming dry N_2_ through a washing bottle containing deionized water using mass flow controllers. Temperature and humidity of the gas stream were verified by a Sensirion SHT2x sensor. The system was allowed to equilibrate for 12 h after each change in temperature and/or humidity.

## 3. Results and Discussion

**Figure 2:**
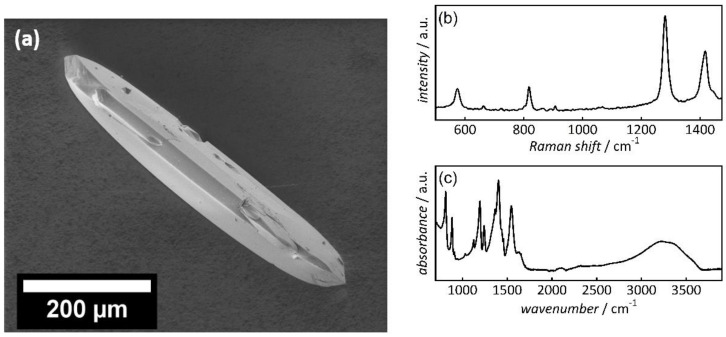
(**a**) Scanning electron microscopic (SEM) image of a Co-MOF-74 crystal (ca. 0.6 mm length); (**b**) Raman spectrum; (**c**) IR spectrum.

The Co-MOF-74 crystals were characterized by SEM, Raman, and IR spectroscopy. Figure 2a shows the SEM image of an exemplary Co-MOF-74 crystal with a representative length of 550 µm and a width around 100 µm. The Raman spectrum (Figure 2b) is in good accordance with previously published MOF-74 Raman data [28]. The peak at 572 cm^−1^ corresponds to the benzene ring vibration (ring deformation mode), the peak at 818 cm^−1^ can be assigned to the C-H bending in the benzene ring, the peak at 1281 cm^−1^ correlates with the C=O stretching, and the O-C-O stretching of the carboxylate group of the organic linker is situated at 1417 cm^−1^ [33]. Figure 2c shows the IR spectrum, with characteristic peaks at 1401 cm^−1^ and 1546 cm^−1^, corresponding to the symmetric and asymmetric ν(COO) stretching of the carboxylic acid group [34]. The measurement was performed under humid air; therefore, the IR spectrum shows a broad band between 2700 cm^−1^ and 3600 cm^−1^, which can be assigned to adsorbed water.

**Figure 3:**
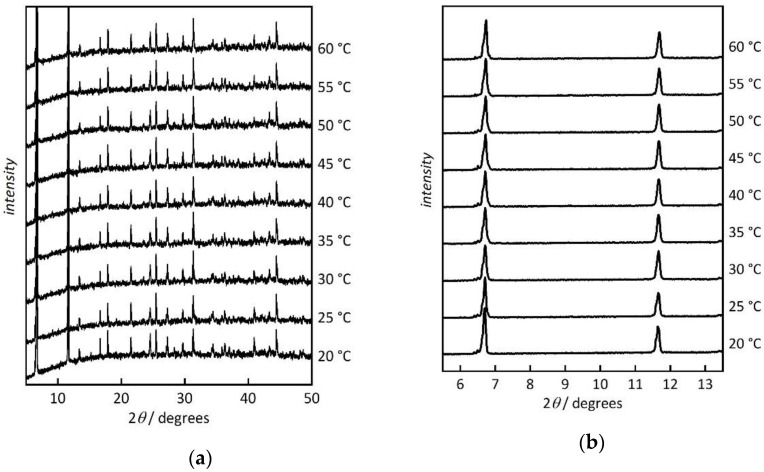
Temperature-resolved powder XRD patterns of Co-MOF-74 (at 93% relative humidity). (**a**) Data range (2*θ*) from 5° to 50°; (**b**) most intense reflections (110 at 6.5°, 300 at 11.6°). Data are shifted vertically for clarity.

Hydrothermal stability is one of basic prerequisites regarding potential application of Co-MOF-74 as a proton-conducting material at elevated temperature and under humid conditions. Therefore, we carried out a temperature-resolved powder XRD analysis at a relative humidity of 93%. Figure 3 shows that the material is stable under the tested conditions. We did not observe any changes in the peak positions over the studied temperature range of 20–60 °C. The diffraction patterns are consistent with literature data [25,35]. Some minor changes in the intensity of the first diffraction peak at 6.7° can be attributed to the different solvent content (here water) occupancy inside the pores, as also reported for other MOFs [36,37].

The proton conductance of Co-MOF-74 was studied by AC impedance analysis. The real part of the complex impedance *Z* corresponds to the resistance *R*, i.e., the reciprocal conductance. The availability of large single crystals made it possible to investigate the material in form of one single crystal, instead of a powder. This eliminates potential difficulties that arise from grain boundary effects or from the impact of surface-adsorbed water in bulk powder or pressed pellets. Most importantly, single-crystal specimens also offer an opportunity to study the anisotropy of proton conductance [30,32,38,39,40,41]. Prior to measurement, the material was dried and activated at 160 °C, to remove adsorbed molecules from the micropores. A color change from red to black is observed upon activation. This effect is reversible; subsequent exposure to air at room temperature (with ambient humidity) turns the color back to red. This observation is attributed to de-coordination/coordination of water ligands to cobalt at the ‘open’ coordination site exposed to the interior of the pores [25].

**Figure 4:**
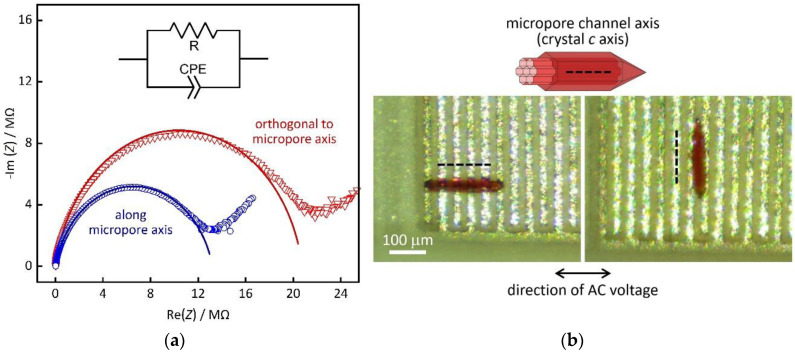
(**a**) Example impedance spectra (Nyquist plots) of a single Co-MOF-74 crystal in two different orientations relative to the contacting electrodes (25 °C, 92% r.h.). Measured data are plotted as scattered points; the lines represent fits by the circuit equivalent shown in the inset. (**b**) Photographs of the crystal on top of the interdigitated electrode structure. (Pt electrodes appear as white lines on the green substrate background) and schematic of pore channel orientation.

**Figure 5:**
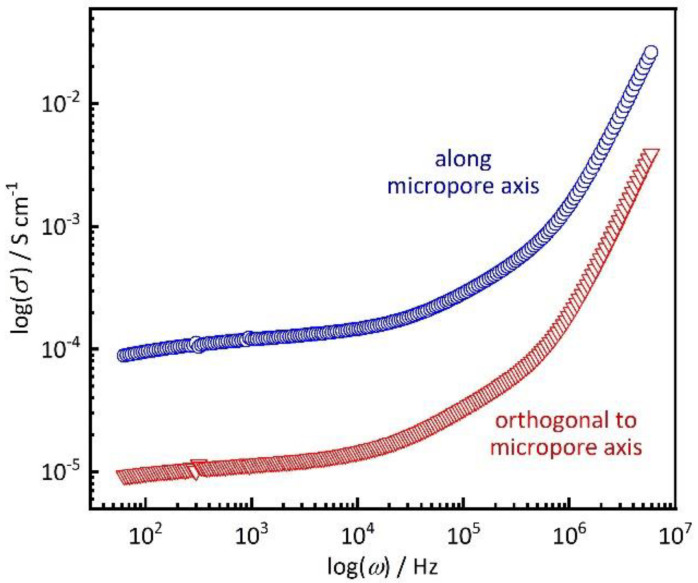
Real part of the conductivity (*σ’*) as a function of frequency (*ω*) for the same data as in Figure 4.

A single crystal was placed on top of an electrode array, as described in the experimental section. It is notable that this method of establishing electrical contact avoids the use of any conductive pastes. Figure 4 shows example Nyquist plots of the impedance *Z* (i.e., imaginary part, -Im(*Z*) vs. real part, Re(*Z*); frequency range from 10 Hz to 1 MHz, applied potential of 0.1 V) of a Co-MOF-74 single crystal at a temperature of 25 °C and relative humidity of 92%. The crystal with an elongated shape was placed in two distinct orientations relative to the electrodes, perpendicular and parallel. Hence, the proton resistance *R* was measured both along the direction of the crystallographic *c* axis (blue) and in an orthogonal direction *a* axis (red). The Nyquist plots exhibit depressed semi-arcs in the high frequency range that can be modeled by a circuit equivalent consisting of a resistor and a parallel constant phase element, as depicted in the inset of Figure 4. The fit parameters, as well as plots of both the real and imaginary parts vs. the frequency, are shown in the Supporting Information section (Table S2 and Figure S3). Even if the proton conductivity turns out not to reach the values of Nafion [9,10], the data still reveal some interesting findings. A relatively high proton conductivity of *σ* = 123 µS cm^−1^ (calculated by Equation (S1), Supplementary Materials section) is observed along the *c* axis, which corresponds to the direction of the micropore channels in MOF-74 [23]. By comparison, the conductivity in the orthogonal direction is only *σ* = 11.7 µS cm^−1^. This is a clear indication that the mobility of the protons is substantially higher along the micropore axis than in the orthogonal direction. Figure 5 shows a plot of the (real part of) the conductivity *σ’* versus the frequency *ω*. The graphs each exhibit a plateau region (near-zero slope in log-log representation) in the frequency region up to ca. 10 kHz. This (nearly) frequency-independent region marks the material-intrinsic conductivity, often referred to as ‘dc conductivity’. The respective plateau values of *σ’* (ca. 100 µS cm^−1^ along the pore channel axis, ca. 10 µS cm^−1^ in orthogonal direction) correspond to the *σ* values calculated from Equation (S1).

**Figure 6:**
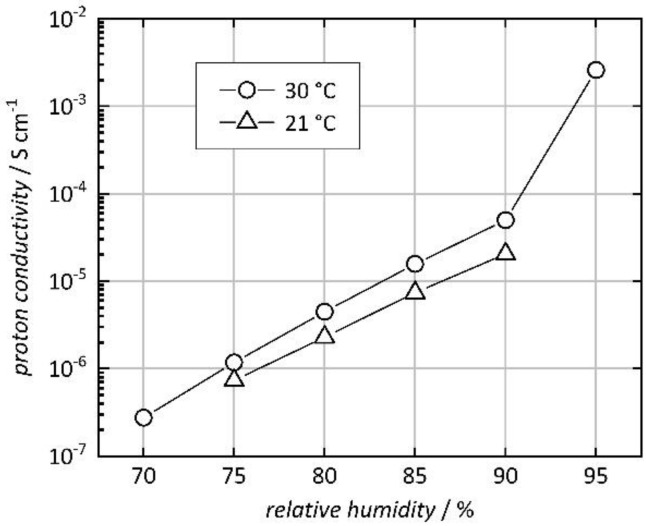
Proton conductivity of a Co-MOF-74 single crystal along the crystallographic *c* axis at 21 °C and 30 °C. (Lines connecting the data points are drawn as a guide to the eye.).

In addition to proton mobility, the protonic conductivity also depends on the number of mobile protons. Since water molecules turn out to be responsible for the proton conduction mechanism (as will be shown below), we varied the relative humidity (r.h.) between 70% and 95%, at 21 °C and 30 °C (along the *c* axis). Figure 6 shows a clear impact of humidity; an exponential increase in conductivity (linear increase at logarithmic scale) is observed up to 90% r.h. (data shown in Table S2, Supplementary Materials section). The very strong increase at 95% r.h. is likely attributable to the formation of a continuous liquid water phase, either inside or outside the pores (bulk water condensation). This effect would be tantamount to ‘short-circuiting’ the sample and, therefore, pose a significant risk to the investigation of intrinsic proton conduction in the MOF material. To avoid such a distortion of the measured data, we used a maximum relative humidity of 90% for all other measurements in this study. (A generally higher conductivity is observed at 30 °C than at 21 °C. The impact of the temperature will be discussed in more detail below.) These findings confirm that the presence of water in the micropores of Co-MOF-74 facilitates protonic conductivity, as frequently observed in MOF materials [30,38,40].

**Figure 7:**
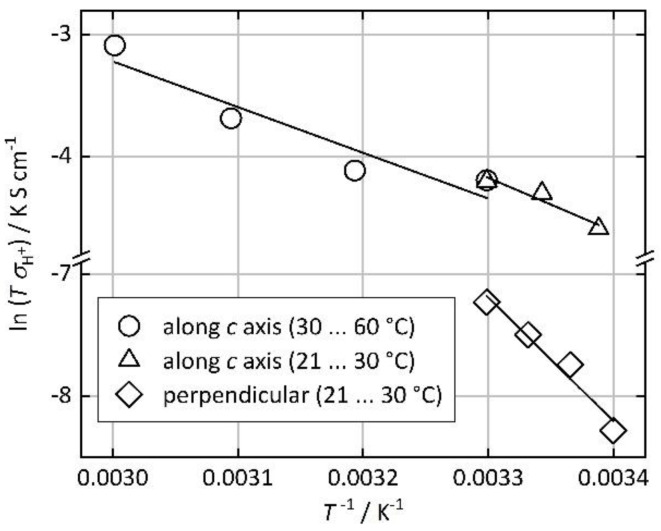
Arrhenius plots of the temperature-dependent proton conductivity of Co-MOF-74 single crystals along the crystallographic *c* axis and in the orthogonal direction (90% r.h.). The lines show the linear regression.

To elucidate the mechanism of proton conduction in more detail, we varied the temperature at a constant relative humidity of 90% (data shown in Table S3, Supplementary Materials section). This offers an opportunity to determine the activation energy *E_A_* for the proton conduction. The conductivity *σ* is related to the temperature *T* by

(1) $$\sigma =\frac{\sigma_{0}}{k_{B}T}\;exp\;\left[\frac{-E_{A}}{k_{B}T}\right]$$

where *σ*_0_ is a material-specific factor and *k_B_* is the Boltzmann constant [13]. A linear regression of ln(*Tσ*) vs. *T*^−1^ (Arrhenius plot) delivers *E_A_* (from the linear slope), as shown in Figure 7. From the data in the temperature range between 21 °C and 30 °C, an activation energy of *E_A_* = 0.37 eV is obtained for the proton conduction in the direction along the micropore axis (*c* axis), while a higher value of *E_A_* = 0.87 eV is found for the orthogonal direction. Further data were measured in the *c* direction between 30 °C and 60 °C, resulting in *E_A_* = 0.32 eV, although, for these measurements, a different crystal was used. These findings strongly suggest that proton conduction in the direction along the micropore axis occurs by a Grotthuss-type mechanism, involving water molecules inside the micropore channels; this is typically associated with activation energies below ca. 0.4 eV [13,42]. In the orthogonal direction, the higher value of *E_A_* indicates a different mechanism, most likely the diffusion of H_3_O^+^ ions. We assume that such diffusion occurs through surface sorbate layers at the crystal surface, rather than inside the crystal.

## 4. Conclusions

In summary, we have measured the proton conductivity in Co-MOF-74 single crystals and found a strong anisotropy. High conductivity (123 µS cm^−1^ at 25 °C) with a low activation energy (0.32 eV) is observed along the direction of the micropore axis (crystallographic *c* axis). In the orthogonal direction, lower conductivity (11.7 µS cm^−1^) and a high activation energy (0.87 eV) are measured. The proton conductivity is strongly humidity-dependent. These findings suggest that proton conduction occurs predominantly through the micropore channels and that water molecules in the channels provide a proton-conducting path by a Grotthuss-type mechanism.

## Supplementary Materials

The following are available online at https://www.mdpi.com/2079-4991/10/7/1263/s1, Figure S1: Microscopic images; Figure S2: Powder XRD diagram; Figure S3: Data vs. frequency: Table S1: Dimensions of single crystals; Table S2: Fit results; Table S3: All measured data; Equation (S1): Calculation of proton conductivity

## References

[1] U.S. Energy Information Administration (2019). International Energy Outlook 2019 (IEO2019).

[2] Fabbri E., Pergolesi D., Traversa E. (2010). Materials challenges toward proton-conducting oxide fuel cells: A critical review. Chem. Soc. Rev..

[3] Wachsman E.D., Lee K.T. (2011). Lowering the Temperature of Solid Oxide Fuel Cells. Science.

[4] Kraytsberg A., Ein-Eli Y. (2014). Review of Advanced Materials for Proton Exchange Membrane Fuel Cells. Energy Fuels.

[5] Gao Z., Mogni L.V., Miller E.C., Railsback J.G., Barnett S.A. (2016). A Perspective on Low-Temperature Solid Oxide Fuel Cells. Energy Environ. Sci..

[6] Majlan E.H., Rohendi D., Dauda W.R.W., Husaini T., Haque M.A. (2018). Electrode for proton exchange membrane fuel cells: A review. Renew. Sust. Energ. Rev..

[7] Breitwieser M., Klingele M., Vierrath S., Zengerle R., Thiele S. (2018). Tailoring the Membrane-Electrode Interface in PEM Fuel Cells: A Review and Perspective on Novel Engineering Approaches. Adv. Energy Mater..

[8] Meyer Q., Zeng Y., Zhao C. (2019). In Situ and Operando Characterization of Proton Exchange Membrane Fuel Cells. Adv. Mater..

[9] Yee R.S.L., Rozendal R.A., Zhang K., Ladewig B.P. (2012). Cost effective cation exchange membranes: A review. Chem. Eng. Res..

[10] Hamrock S.J., Yandrasits M.A. (2006). Proton Exchange Membranes for Fuel Cell Applications. J. Macromol. Sci. Part C Polym. Rev..

[11] Shimizu G.K.H., Taylor J.M., Kim S. (2013). Proton Conduction with Metal-Organic Frameworks. Science.

[12] Yamada T., Otsubo K., Makiura R., Kitagawa H. (2013). Designer coordination polymers: Dimensional crossover architectures and proton conduction. Chem. Soc. Rev..

[13] Ramaswamy P., Wong N.E., Shimizu G.K.H. (2014). MOFs as proton conductors—Challenges and opportunities. Chem. Soc. Rev..

[14] Tominaka S., Cheetham A.K. (2014). Intrinsic and extrinsic proton conductivity in metal-organic frameworks. RSC Adv..

[15] Nagarkar S.S., Unni S.M., Sharma A., Kurungot S., Ghosh S.K. (2014). Two-in-One: Inherent Anhydrous and Water-Assisted High Proton Conduction in a 3D Metal-Organic Framework. Angew. Chem. Int. Ed..

[16] Moghadam P.Z., Li A., Wiggin S.B., Tao A., Maloney A.G.P., Wood P.A., Ward S.C., Fairen-Jimenez D. (2017). Development of a Cambridge Structural Database Subset: A Collection of Metal-Organic Frameworks for Past, Present, and Future. Chem. Mater..

[17] Yin Z., Wan S., Yang J., Kurmoo M., Zeng M.-H. (2019). Recent advances in post-synthetic modification of metal–organic frameworks: New types and tandem reactions. Coord. Chem. Rev..

[18] Kitagawa S., Kitaura R., Noro S. (2004). Functional porous coordination polymers. Angew. Chem. Int. Ed..

[19] Ferey G. (2008). Hybrid porous solids: Past, present, future. Chem. Soc. Rev..

[20] Tranchemontagne D.J., Mendoza-Cortés J.L., O’Keeffe M., Yaghi O.M. (2009). Secondary building units, nets and bonding in the chemistry of metal–organic frameworks. Chem. Soc. Rev..

[21] Czaja A.U., Trukhan N., Müller U. (2009). Industrial applications of metal–organic frameworks. Chem. Soc. Rev..

[22] Furukawa H., Cordova K.E., O’Keeffe M., Yaghi O.M. (2013). The Chemistry and Applications of Metal-Organic Frameworks. Science.

[23] Dietzel P.D.C., Morita Y., Blom R., Fjellvåg H. (2005). An In Situ High-Temperature Single-Crystal Investigation of a Dehydrated Metal-Organic Framework Compound and Field-Induced Magnetization of One-Dimensional Metal-Oxygen Chains. Angew. Chem. Int. Ed..

[24] Rosi N.L., Kim J., Eddaoudi M., Chen B., O’Keeffe M., Yaghi O.M. (2005). Rod Packings and Metal−Organic Frameworks Constructed from Rod-Shaped Secondary Building Units. J. Am. Chem. Soc..

[25] Chmelik C., Mundstock A., Dietzel P.D.C., Caro J. (2014). Idiosyncrasies of Co_2_(dhtp): In situ-annealing by methanol. Microporous Mesoporous Mater..

[26] Valvekens P., Vandichel M., Waroquier M., Van Speybroeck V., De Vos D. (2014). Metal-dioxidoterephthalate MOFs of the MOF-74 type: Microporous basic catalysts with well-defined active sites. J. Catal..

[27] Dietzel P.D.C., Besikiotis V., Blom R. (2009). Application of metal-organic frameworks with coordinatively unsaturated metal sites in storage and separation of methane and carbon dioxide. J. Mater. Chem..

[28] Strauss I., Mundstock A., Hinrichs D., Himstedt R., Knebel A., Reinhardt C., Dorfs D., Caro J. (2018). The Interaction of Guest Molecules with Co-MOF-74: A Vis/NIR and Raman Approach. Angew. Chemie Int. Ed..

[29] Strauss I., Mundstock A., Treger M., Lange K., Hwang S., Chmelik C., Rusch P., Bigall N.C., Pichler T., Shiozawa H. (2019). Metal–Organic Framework Co-MOF-74-Based Host–Guest Composites for Resistive Gas Sensing. ACS Appl. Mater. Interfaces.

[30] Hwang S., Lee E.J., Song D., Jeong N.C. (2018). High Proton Mobility with High Directionality in Isolated Channels of MOF-74. ACS Appl. Mater. Interfaces.

[31] Momma K., Izumi F. (2011). VESTA 3 for three-dimensional visualization of crystal, volumetric and morphology data. J. Appl. Crystallogr..

[32] Javed A., Wagner T., Wöhlbrandt S., Stock N., Tiemann M. (2020). Proton Conduction in a Single Crystal of a Phosphonato-Sulfonate-Based Coordination Polymer: Mechanistic Insight. ChemPhysChem.

[33] Bonino F., Chavan S., Vitillo J.G., Groppo E., Agostini G., Lamberti C., Dietzel P.D.C., Prestipino C., Bordiga S. (2008). Local Structure of CPO-27-Ni Metallorganic Framework upon Dehydration and Coordination of NO. Chem. Mater..

[34] Zhao Z., Zuhra Z., Qin L., Zhou Y., Zhang L., Tang F., Mu C. (2018). Confinement of microporous MOF-74(Ni) within mesoporous γ-Al_2_O_3_ beads for excellent ultra-deep and selective adsorptive desulfurization performance. Fuel Process. Technol..

[35] Dietzel P.D.C., Johnsen R.E., Blom R., Fjellvåg H. (2008). Structural Changes and Coordinatively Unsaturated Metal Atoms on Dehydration of Honeycomb Analogous Microporous Metal–Organic Frameworks. Chem. Eur. J..

[36] Canivet J., Fateeva A., Guo Y., Coasne B., Farrusseng D. (2014). Water adsorption in MOFs: Fundamentals and applications. Chem. Soc. Rev..

[37] Øien-Ødegaard S., Shearer G.C., Wragg D.S., Lillerud K.P. (2017). Pitfalls in metal–organic framework crystallography: Towards more accurate crystal structures. Chem. Soc. Rev..

[38] Yoon M., Suh K., Kim H., Kim Y., Selvapalam N., Kim K. (2011). High and Highly Anisotropic Proton Conductivity in Organic Molecular Porous Materials. Angew. Chem. Int. Ed..

[39] Tominaka S., Henke S., Cheetham A.K. (2013). Coordination polymers of alkali metal trithiocyanurates: Structure determinations and ionic conductivity measurements using single crystals. Cryst. Eng. Comm..

[40] Li R., Wang S.H., Chen X.X., Lu J., Fu Z.H., Li Y., Xu G., Zheng F.K., Guo G.C. (2017). Highly Anisotropic and Water Molecule-Dependent Proton Conductivity in a 2D Homochiral Copper(II) Metal-Organic Framework. Chem. Mater..

[41] Bunzen H., Javed A., Klawinski D., Lamp A., Grzywa M., Kalytta-Mewes A., Tiemann M., von Nidda H.-A.K., Wagner T., Volkmer D. (2019). Anisotropic Water-Mediated Proton Conductivity in Large Iron(II) Metal–Organic Framework Single Crystals for Proton-Exchange Membrane Fuel Cells. ACS Appl. Nano Mater..

[42] Kreuer K.-D. (1996). Proton Conductivity: Materials and Applications. Chem. Mater..

