# Supplementary material for: Humidity-Mediated Anisotropic Proton Conductivity through the 1D Channels of Co-MOF-74
Source: Nanomaterials (Basel). 2020 Jun 28;10(7):1263. doi: 10.3390/nano10071263 (PMC7407165; doi:10.3390/nano10071263)
Supplement: Supplementary file 1 [file nanomaterials-10-01263-s001.pdf]

Humidity-mediated Anisotropic Proton Conductivity through the 1D Channels of Co-MOF-74

Supplementary Material

Ali Javed [1], Ina Strauss [2], Hana Bunzen [3], Jürgen Caro [2], and Michael Tiemann [1]

[1] Department of Chemistry, Paderborn University, 33098 Paderborn, Germany

[2] Institute of Physical Chemistry and Electrochemistry, Leibniz University Hannover, 30167 Hannover, Germany

[3] Institute of Physics, University of Augsburg, 86159 Augsburg, Germany

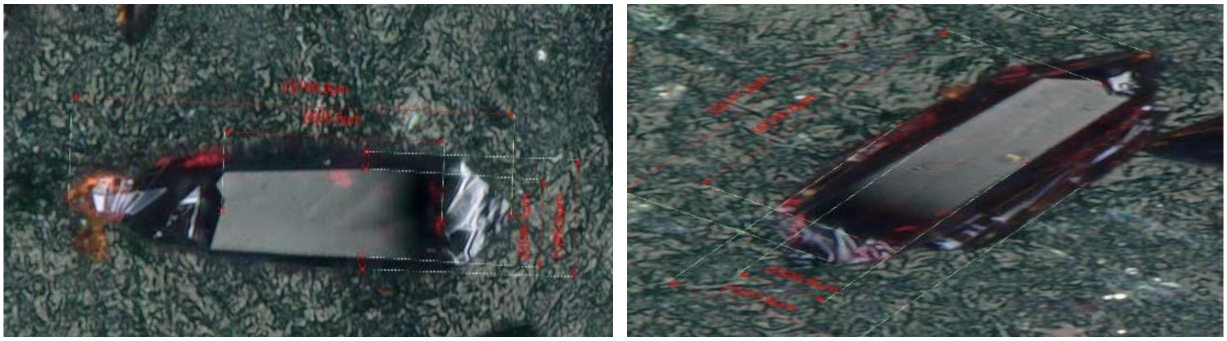

Figure S1. Microscopic images of two Co-MOF-74 crystals for the evaluation of the contact area in equation S1.

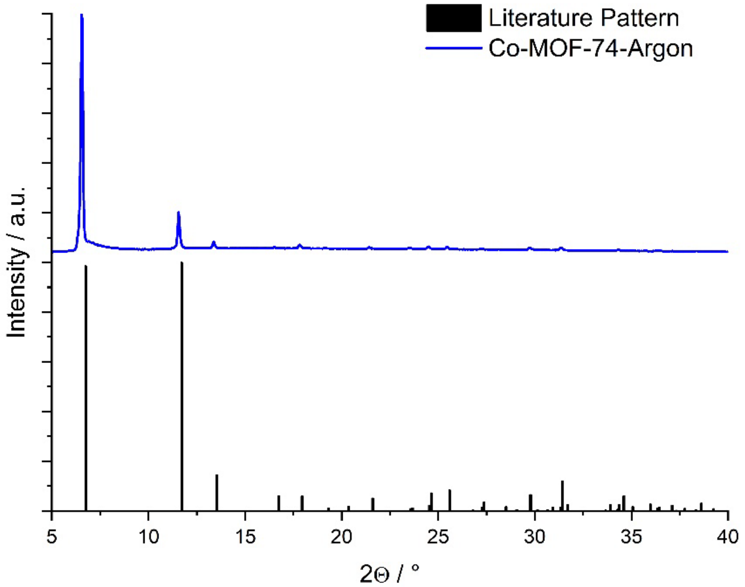

Figure S2. Experimental and simulated powder XRD pattern of Co-MOF-74 [1].

[1] Strauss, I.; Mundstock, A.; Hinrichs, D.; Himstedt, R.; Knebel, A.; Reinhardt, C.; Dorfs, D.; Caro, J. *Angew. Chemie Int. Ed.* **2018**, *57*, 7434–7439.

Table S1. Dimensions of Co-MOF-74 single crystals and contact lengths/widths with underlaying flat substrate, determined from Figure S1. The values are used as correcting factors for the assessment of the contact area in equation S1.

crystal #	*length*	*contact length*	*width*	*contact width*
1	45.6 μm	34.1 μm (74.8%)	190.9 μm	95 μm (49.8%)
2	141.2 μm	105.0 μm (74.8%)	57 μm	26 μm (45.6%)

Equation S1. Calculation of the proton conductivity σ from the measured proton resistance R and the contact area with the Pt electrodes (A: contact area between sample and the Pt electrode array; n: number of spacings between electrodes, D: inter-electrode distance (20 μm), d: electrode width (20 μm), a: width of crystal) [2].

$$\sigma = n\cdot(D / (R\cdot A)) = n\cdot(D / (R\cdot a\cdot d)) \qquad \text{(S1)}$$

Table S2. Fit results for the spectra in Figure 4.

	along micropore axis	orthogonal to axis
χ^2	0.015	0.014
CPE-T	1.8753×10^{-11}	1.4715×10^{-11}
CPE-P	0.88579	0.89969
R / Ω	1.278×10^{7}	2.072×10^{7}

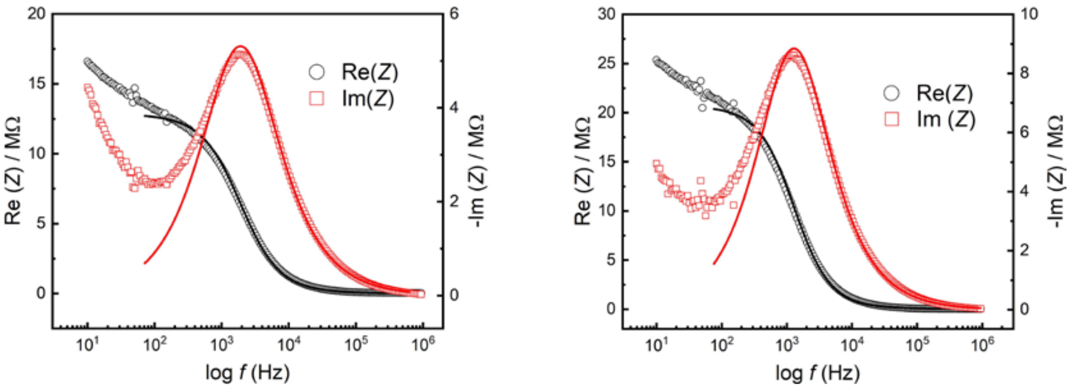

Figure S3. Real and imaginary parts of the impedance *vs.* frequency of a single Co-MOF-74 crystal in two different orientations relative to the contacting electrodes (25 °C, 92% r.h.): along (left) and orthogonal (right) to the micropore axis.

[2] Javed, A.; Wagner, T.; Wöhlbrandt, S.; Stock, N.; Tiemann, M. *ChemPhysChem* **2020**, *21*, 605–609.

Table S3. All measured proton conductivity values of Co-MOF-74 crystals under variable conditions.

**sample name**	**proton current direction**	***T* / °C**	**r.h. / %**	***proton conductivity* σ / µS cm^{-1}**
Co-74_1	=	25	92	123
	≠	25	92	11.7
Co-74_2	=	21	75	0.732
	=	21	80	2.29
	=	21	85	7.38
	=	21	90	20.4
Co-74_3	=	22	90	34.4
	=	26	90	45.5
	=	30	90	49.6
Co-74_4	≠	21	90	0.862
	≠	24	90	1.46
	≠	27	90	1.85
	≠	30	90	2.40
Co-74_5	=	30	70	0.27
	=	30	75	1.18
	=	30	80	4.48
	=	30	85	15.7
	=	30	90	49.8
	(=)	(30)	(95)	(2590)[2]
	=	40	90	52.3
	=	50	90	77.7
	=	60	90	137

[1] "=": along *c* axis; "≠": orthogonal [2] (presumed bulk water condensation)
